# Protective CD8+ T lymphocytes in Primates Immunized with Malaria Sporozoites

**DOI:** 10.1371/journal.pone.0031247

**Published:** 2012-02-15

**Authors:** Walter R. Weiss, Chengyong George Jiang

**Affiliations:** Infectious Disease Department, Naval Medical Research Center, Silver Spring, Maryland, United States of America; Museum National d'Histoire Naturelle, France

## Abstract

Live attenuated malaria vaccines are more potent than the recombinant protein, bacterial or viral platform vaccines that have been tested, and an attenuated sporozoite vaccine against falciparum malaria is being developed for humans. In mice, attenuated malaria sporozoite vaccines induce CD8^+^ T cells that kill parasites developing in the liver. We were curious to know if CD8^+^ T cells were also important in protecting primates against malaria. We immunized 9 rhesus monkeys with radiation attenuated *Plasmodium knowlesi* sporozoites, and found that 5 did not develop blood stage infections after challenge with live sporozoites. We then injected 4 of these protected monkeys with cM-T807, a monoclonal antibody to the CD8 molecule which depletes T cells. The fifth monkey received equivalent doses of normal IgG. In 3 of the 4 monkeys receiving cM-T807 circulating CD8^+^ T cells were profoundly depleted. When re-challenged with live sporozoites all 3 of these depleted animals developed blood stage malaria. The fourth monkey receiving cM-T807 retained many circulating CD8^+^ T cells. This monkey, and the vaccinated monkey receiving normal IgG, did not develop blood stage malaria at re-challenge with live sporozoites. Animals were treated with antimalarial drugs and rested for 4 months. During this interval CD8^+^ T cells re-appeared in the circulation of the depleted monkeys. When all vaccinated animals received a third challenge with live sporozoites, all 5 monkeys were once again protected and did not develop blood stage malaria infections. These data indicate that CD8^+^ T cells are important effector cells protecting monkeys against malaria sporozoite infection. We believe that malaria vaccines which induce effector CD8+ T cells in humans will have the best chance of protecting against malaria.

## Introduction

Mice [1], [2], monkeys [3], and humans [4] can be protected against malaria infection by live attenuated malaria sporozoite vaccines, and a commercial attenuated sporozoite vaccine against falciparum malaria is being developed [5]. In mice, attenuated sporozoite vaccines induce CD8^+^ T cells which kill parasites developing in the liver. Two studies have found that mice depleted of CD8^+^ T cells are no longer protected by attenuated sporozoite vaccines [6], [7]. However, a third study using a different mouse/malaria combination did not confirm this finding, indicating that other immune effectors might be involved in protecting mice [8]. In primates and humans protected by attenuated sporozoite vaccines the immune responses which kill developing parasites have not been identified. In this paper we have protected monkeys with an attenuated sporozoite vaccine, and show that this protection disappears when animals are treated with a monoclonal antibody to CD8 that depletes circulating lymphocytes [9]. In addition, we find that as the effects of the monoclonal antibody wane and CD8^+^ lymphocytes reappear, monkeys regain the protective immunity they had lost. Since in both mammalian models (mice and monkeys) CD8^+^ effector cells play a key role in protection from live attenuated sporozoite vaccines, it is likely that CD8^+^ cells are important immune effector cells against human malaria as well.

## Results

### Immunization and 1^st^ Challenge

A total of 9 rhesus monkeys were immunized with irradiated *Plasmodium knowlesi* (Pk) sporozoites in two cohorts of 5 and 4 animals. Each cohort of vaccinated monkeys and 5 naïve controls were challenged with infectious Pk sporozoites. All control monkeys developed blood stage infections after challenge. Two vaccinated animals in each of the cohorts became infected (data not shown) but a total of five vaccinated animals were protected, 3 from Cohort 1 and 2 from Cohort 2. (Table 1, monkeys A–E).

**Table 1:** Effect of anti-CD8 Mab treatment in monkeys protected by the irradiated sporozoite vaccine.

	Monkey	Vaccine	1st Challenge Infected	% CD8^+^ before Ab	Ab before 2nd Challenge	% CD8^+^ after Ab	2nd Challenge Infected	% CD8^+^ before 3rd Challenge	3rd Challenge Infected
Cohort 1	A	Irr.Spz	No	29	IgG	37	No	39	No
	B	Irr.Spz	No	38	aCD8	<1	Yes	24	No
	C	Irr.Spz	No	39	aCD8	14	No	33	No
	Controls	none	5/5*Yes				3/3* Yes		3/3** Yes
Cohort 2	D	Irr.Spz	No	30	aCD8	<1	Yes	15	No
	E	Irr.Spz	No	40	aCD8	<1	Yes	9	No
	Controls	none	5/5* Yes				8/8* Yes		4/4** Yes

In the two Cohorts, a total of five monkeys vaccinated with irradiated Pk sporozoites were protected in their 1^st^ Challenge with malaria sporozoites. Before their 2^nd^ Challenge, Monkey A received control IgG while monkeys B–E received anti-CD8 Mab. Monkeys B, D, and E experienced a profound drop in circulating CD3**^+^**CD8**^+^** T cells and became infected when given a 2^nd^ Challenge with sporozoites. Monkey A was protected. Monkey C retained some CD3**^+^**CD8**^+^** T cells and was also protected. After the 2^nd^ Challenge, monkeys B, D, and E and control monkeys were drug treated and rested for 4 months before their 3^rd^ Challenge. At the time of the 3^rd^ Challenge circulating CD3**^+^**CD8**^+^** T cells had returned, and all 5 vaccinated monkeys were again protected.

*Control monkeys in the 1^st^ and 2^nd^ Challenges were malaria naïve.

**Control monkeys in the 3^rd^ Challenge had been infected once in the 2^nd^ Challenge.

In blood taken two weeks after the last vaccination, we could not detect CD4+ or CD8+ T cells reactive to the Pk Circumsporozoite Protein (PkCSP) or Pk Apical Merozoite Antigen −1 (PkAMA1) using assays for Interferon-γ (data not shown) [10]. However, all vaccinated monkeys had strong serum IFAT titers against Pk sporozoites (data not shown) [11].

### Anti-CD8 treatment and 2^nd^ Challenge Cohort 1

Two months after their 1st Challenge, monkeys B and C were treated with cM-T807, a humanized mouse Mab to CD8 alpha chain [9]. Monkey A received control human IgG. After antibody treatments, monkeys A, B and and C and 3 new controls monkeys received their 2^nd^ Challenge with infectious Pk sporozoites.

All 3 controls developed blood stage infections (Table 1). Monkey A (receiving human IgG) was protected in the 2^nd^ Challenge. Monkey B developed blood stage parasites. The circulating CD3^+^CD8^+^ lymphocytes in this animal dropped to less than 1% of total CD3+ lymphocytes after receiving anti-CD8 Mab. However Monkey C's circulating CD3^+^CD8^+^ circulating lymphocytes only dropped from 39% to 14%, and Monkey C remained protected. All monkeys with blood stage infections in the 2^nd^ Challenge (2 vaccinated and 3 controls) had their first parasites detected in the blood between days 8–12 after challenge. Infected animals were treated with artesunate and chloroquine and rested until their 3^rd^ Challenge.

### Anti-CD8 treatment and 2^nd^ Challenge Cohort 2

Two months after their 1^st^ Challenge, monkeys D and E were treated with Mab cM-T807. Both monkeys and 8 new controls then received their 2^nd^ Challenge with infectious Pk sporozoites.

All 8 control monkeys developed blood stage infections (Table 1). Monkeys D and E also were infected in the 2^nd^ Challenge. The circulating CD3^+^CD8^+^ lymphocytes in these animals dropped to less than 1% of total CD3+ lymphocytes after receiving anti-CD8 Mab. All monkeys had their first parasites detected in the blood between days 8–12 after challenge. They were treated with artesunate and chloroquine and rested until their 3^rd^ Challenge.

### 3^rd^ Challenge

Four months after their 2^nd^ Challenges, the monkeys from Cohorts 1 and 2 received a 3^rd^ Challenge with Pk sporozoites. From Cohort 1 this included 3 vaccinated monkeys A, B and C and 3 controls from the 2^nd^ challenge. In Cohort 2, this included 2 vaccinated monkeys D and E and 4 controls from the 2^nd^ challenge. At this time, CD3^+^CD8^+^ circulating lymphocytes had reappeared (Table 1). All 7 controls developed blood stage parasites while none of the 5 vaccinated monkeys became infected.

## Discussion

In three vaccinated and protected monkeys, Mab treatment successfully removed circulating CD8^+^ lymphocytes, and all three of these animals lost protection from an attenuated sporozoite vaccine. As CD8^+^ T cells re-appeared in these monkeys, all three regained protection against malaria infection. In rhesus monkeys, the CD8 alpha-chain is present at high levels on CD8^+^ T cells and some NK cells, and at low levels on some B cells, CD4^+^ CD8^+^ double positive T cells and dendritic cells (personal communication from Louis Picker). While any of these cell types might have been affected by the anti-CD8 antibody treatments, we think CD8^+^ T cells are most likely involved in killing malaria parasites in the liver after sporozoite infection. Return of protection after anti-CD8 Mab treatment might be due to persistent malaria antigen immunizing the regrown T cells [12]. Alternatively, CD8 Mab may have blocked function of CD8^+^ cells in the liver without eliminating them, and with time these cells may have regained their activity.

We were surprised that a larger fraction of vaccinated monkeys were not protected against malaria infection. In the only previous paper describing immunization of rhesus monkeys with Pk sporozoites [3], 3 animals were given a total of 3 million irradiated parasites IV, and 2/3 were protected when challenged with live sporozoites. In our 5 monkeys in Cohort 1, we gave a total of 2.8 million sporozoites IV and 3/5 were protected in their 1^st^ Challenge. In Cohort 2, we attempted to protect all 4 monkeys by increasing the immunizing dose to 5.2 million sporozoites. However in Cohort 2, only 2/4 animals were protected in their 1^st^ Challenge with live sporozoites. Overall 5/9 vaccinated monkeys were protected, similar to results in the earlier publication. This contrasts with the human data on immunization with P. falciparum sporozoites, where over 90% of human volunteers have been protected [4].

There are several possible explanations for the poorer efficacy of the irradiated sporzoite vaccine in monkeys as compared to humans. Firstly, we gave irradiated Pk sporozoites as they became available, and our dose size varied considerably which may have led to less than optimal vaccinations (Table 2 gives the sporozoite doses for each cohort). Secondly, it is possible that Pk sporozoites are less immunogenic or more infectious than P. falciparum sporozoites. Thirdly, human volunteers are immunized and challenged by the bite of irradiated mosquitoes. In our primate experiments, we gave sporozoites IV for both immunizations and challenges. It may be that IV parasites induce different immune responses from those generated by mosquito bite to the skin [13]. Alternatively, the challenge with live IV sporozoites, bypassing the skin, may have skipped over an important site of parasite killing [14].

**Table 2:** Sporozoite immunization schedule.

	Dose	Month	Irradiated Sporozoites (thousands)
Cohort 1	1	1	300
	2	4	366
	3	7	67
	4	17	24
	5	18	81
	6	20	666
	7	22	333
	8	24	1000
	Total sporozoites		2837
Cohort 2	1	1	100
	2	2	250
	3	5	170
	4	6	500
	5	9	180
	6	13	4000
	Total sporozoites		5200

The genetic diversity of the rhesus monkeys may explain the low vaccine efficacy. Studies of irradiated sporozoite vaccines in inbred mice typically use the IV route for immunization and challenge. Genetically defined mouse strains vary dramatically in the number of live sporozoites required to infect naïve animals [15], as well as the number of irradiated sporozoites required to protect animals against infection [16], [17]. It may be that immunogenetic effects are more pronounced when sporozoites are given IV, It would be interesting to vaccinate and challenge monkeys with sporozoites by mosquito bite, to see if vaccine efficacy was closer to that in humans.

We were not able to detect T cell responses to two malaria sporozoite antigens, PkCSP and PkAMA-1, in the venous blood of our monkeys vaccinated with irradiated sporozoites. In humans, there is evidence that PfCSP is a protective target antigen. PfCSP is the basis of the RTS,S vaccine which protects against malaria in lab and field studies. However, several studies in murine malaria have indicated that other malaria antigens may be more important targets of immunity than CSP [18], [19], [20], [21]. Prior to this study, there was no previously published data on immune responses to PkCSP in monkeys vaccinated with attenuated sporozoites. PkAMA1 was first described as a blood stage merozoite antigen, but AMA1 expression has been found in sporozoites of P. falciparum [22]. Recent human trials from our laboratory have shown that human immunized with PfCSP and PfAMA1 can be protected against infection by sporozoites, while volunteers immunized with PfCSP alone are not protected (unpublished data). The choice of PkCSP and PkAMA1 to monitor immune responses was reasonable, but we do not believe that these antigens are necessarily the primary targets of the protective immune responses

In this protocol, we did not test lymphocytes from the livers of our immunized monkeys for immune responses. In our previous primate studies using recombinant Pk vaccines, we tested peripheral blood and readily detected CD4^+^ T cell responses to PkCSP and PkAMA1 but rarely CD8^+^ T cell responses [10], [23]. In human volunteers immunized with irradiated sporozoites, T cell responses are low in peripheral blood [24], [25]. Recently, Epstein et al. [26] have immunized rhesus monkeys with irradiated P. falciparum sporozoites given IV, and they discovered strong CD4^+^ and CD8^+^ T cell responses in liver lymphocytes but weaker T cell responses in circulating lymphocytes. In the same manner, our vaccinated monkeys may have protective T cells in their liver compartment while lacking them in the blood.

If CD8+ T cells are important in malaria immunity in monkeys, these effector cells may well play an important role in protecting humans against malaria infection. Recombinant vaccines that induce CD8+ T cell immunity should be vigorously investigated [27].

## Methods

### Ethics Statement

We used adult *Macaca mulatta* (rhesus) monkeys bred in the US from Indian stock and maintained in a facility accredited by the Association for Assessment and Accreditation of Laboratory Animal Care (AAALC). The experiments were conducted in compliance with the Animal Welfare Act and in accordance with the principles set forth in the “Guide for the Care and Use of Laboratory Animals,” Institute of Laboratory Animals Resources, National Research Council, National Academy Press, 1996. All experiments were approved by the Institutional Animal Care and Use Committee, Walter Reed Army Institute of Research/Naval Medical Research Center, Silver Spring Maryland, USA. To ameliorate suffering, all animals with malaria infections were closely monitored and treated with anti-malarial drugs.

### Malaria sporozoites for challenge and vaccination

*Plasmodium knowlesi* H strain sporozoites were produced in *Anopheles dirus* mosquitoes. Sporozoites were dissected into M199 medium (Sigma-Aldrich Inc., St. Louis, MO) with 5% normal rhesus monkey serum. For malaria challenge, 100 Pk sporozoites were injected IV in a 1 ml volume. For malaria vaccination, sporozoites received 150 Gy from a CS-137 source before being injected IV in a 1 ml volume.

### Experimental Cohorts 1 and 2

These experiments were carried out in two cohorts of monkeys. Cohort 1 included 5 monkeys for immunization, antibody treatments, and challenges. After the completion of all work on Cohort 1, the experiment was repeated in Cohort 2 with 4 monkeys.

### Immunization schedules and protection

Cohort 1 included 5 monkeys that received a total of 2.8 million sporozoites in 10 doses over 24 months. Three of these animals (Table 1 monkeys A, B, and C) were protected in their 1^st^ Challenge with Pk sporozoites and two developed blood stage infections. Cohort 2 included 4 monkeys that received a total of 5.2 million sporozoites in 6 doses over 12 months. Two of these monkeys (Table 1 monkeys D and E) were protected in their 1^st^ Challenge with Pk sporozoites and two developed blood stage infections. Table 2 gives the details of sporozoite immunization for Cohorts 1 and 2.

### Monitoring and treatment of malaria infections

After sporozoite challenge, from day 6 to day 30 blood was obtained by skin prick for thin and thick film malaria slides. After Giemsa staining, blood was examined under ×1000 magnification until 20,000 red cells on thin film were examined. Negative results were confirmed reading the thick film slides. Protection was defined as the complete absence of malaria parasites on all days. Infected animals were treated when parasitemias reached 2% with artesunate (5 mg/kg single dose) and chloroquine (45 mg/kg in divided doses over 5 days). Animals were monitored for 30 days to ensure effectiveness of treatment in clearing malaria infections.

### In vivo CD8 depletion

Monkeys were injected with the mouse-human chimeric anti-human CD8 alpha chain monoclonal antibody, cM-T807 [9] (Centocor, Inc, Horsham, PA). Monkey A received control injection with Human IgG (Sigma-Aldrich Inc., St. Louis, MO). In Cohort 1, 10 mg/kg antibody was injected SC 7 days before sporozoite challenge, and 5 mg/kg was injected IV 4 days and 1 day before and 2 days after challenge. In Cohort 2, 50 mg/kg antibody was injected in a single IV dose 3 days before challenge. Dosing was the same for cM-T807 and control IgG.

### Monitoring of blood lymphocyte subsets

Lymphocyte subsets in venous blood samples were determined using flow cytometry. Reagents were from BD Biosciences (San Jose, California, USA): anti-CD3apc-cy7, anti-CD4 percp-cy5.5, anti-CD8PE, anti-CD20 FITC, and anti-CD16 FITC. We used a LSR-II flow cytometer and DIVA software (BD Biosciences).

### Measurement of immune responses after vaccination

Blood lymphocytes were tested by flow cytometry for interferon-γ production when restimulated in vitro using overlapping 15 amino acid peptide sequences from the Pk circumsporozoite protein and Pk apical merozoite antigen-1 (42 kD) antigens as previously described [10]. Assessment of serum antibody to Pk sporozoites was by IFAT [11].
